# Functional Selectivity and Antidepressant Activity of Serotonin 1A Receptor Ligands

**DOI:** 10.3390/ijms160818474

**Published:** 2015-08-07

**Authors:** Zdzisław Chilmonczyk, Andrzej Jacek Bojarski, Andrzej Pilc, Ingebrigt Sylte

**Affiliations:** 1 National Medicines Institute, Chełmska 30/34, 00-725 Warszawa, Poland; 2 Institute of Nursing and Health Sciences, University of Rzeszów, W. Kopisto 2A, 35-310 Rzeszów, Poland; 3 Institute of Pharmacology, Polish Academy of Sciences, Smetna 12, 31-343 Kraków, Poland; E-Mails: bojarski@if-pan.krakow.pl (A.J.B.); nfpilc@cyf-kr.edu.pl (A.P.); 4 Faculty of Health Sciences, University of Tromsø—The Arctic University of Norway, No-9037 Tromsø, Norway; E-Mail: ingebrigt.sylte@uit.no

**Keywords:** serotonin 1A receptors, receptor trafficking, antidepressant activity

## Abstract

Serotonin (5-HT) is a monoamine neurotransmitter that plays an important role in physiological functions. 5-HT has been implicated in sleep, feeding, sexual behavior, temperature regulation, pain, and cognition as well as in pathological states including disorders connected to mood, anxiety, psychosis and pain. 5-HT_1A_ receptors have for a long time been considered as an interesting target for the action of antidepressant drugs. It was postulated that postsynaptic 5-HT_1A_ agonists could form a new class of antidepressant drugs, and mixed 5-HT_1A_ receptor ligands/serotonin transporter (SERT) inhibitors seem to possess an interesting pharmacological profile. It should, however, be noted that 5-HT_1A_ receptors can activate several different biochemical pathways and signal through both G protein-dependent and G protein-independent pathways. The variables that affect the multiplicity of 5-HT_1A_ receptor signaling pathways would thus result from the summation of effects specific to the host cell milieu. Moreover, receptor trafficking appears different at pre- and postsynaptic sites. It should also be noted that the 5-HT_1A_ receptor cooperates with other signal transduction systems (like the 5-HT_1B_ or 5-HT_2A/2B/2C_ receptors, the GABAergic and the glutaminergic systems), which also contribute to its antidepressant and/or anxiolytic activity. Thus identifying brain specific molecular targets for 5-HT_1A_ receptor ligands may result in a better targeting, raising a hope for more effective medicines for various pathologies.

## 1. Introduction

Serotonin is a monoamine neurotransmitter that plays an important role in physiological functions, such as sleep, feeding, sexual behavior, temperature regulation, pain, and cognition, as well as in pathological states including mood disorders, anxiety disorders, psychosis, and pain disorders. Medications that increase the level of 5-HT, such as the selective serotonin reuptake inhibitors, are used as treatments of depression and anxiety. The seven 5-HT receptor classes consist of 5-HT_1_, 5-HT_2_, 5-HT_3_, 5-HT_4_, 5-HT_5_, 5-HT_6_, 5-HT_7_, which are further subdivided into 14 receptor subclasses. All of these receptors—except for 5-HT_3_ receptor class, which belongs to the family of ligand-gated ionic channels and is permeable to Na^+^, K^+^, Ca^2+^ (and other cations)—belong to the superfamily of seven-transmembrane-domain, G protein-coupled receptors (GPCRs). For serotonin GPCRs, three main types of primary coupling to G proteins have been described. The 5-HT_1A_ receptors activate G_i_/G_o_ proteins, the 5-HT_2A_ receptors activate G_q_/G_11_, and the 5-HT_4_, 5-HT_6_, and 5-HT_7_ activate G_s_ [1].

5-HT neurons show a number of anatomical and physiological characteristics, some of which are shared by noradrenergic neurons [2,3,4]:

- Low number of neurons: 250,000 5-HT neurons in the human brain (out of a total of 10^11^).
- Innervation of the whole neuraxis with extensive axon branching (4106 nerve terminals/mm^3^ in neocortex).
- Slow and regular discharge (pace maker neurons): strong homeostasis.
- Neuronal activity dependent on sleep-wake cycles (REM-off neurons).
- Very sensitive to self-inhibition through activation of 5-HT_1A_ autoreceptors.
- Rich neurochemistry: 14 different postsynaptic receptors.
- Implication in a large number of physiological functions.
- Mutual control with monoaminergic cell groups.

5-HT neuronal activity is tightly controlled by several afferent pathways, including glutamatergic inputs from the forebrain (e.g., prefrontal cortex, PFC), monoaminergic, peptidergic and local GABAergic inputs [5]. Overall, this means that serotonergic activity and 5-HT release in the forebrain is very tightly controlled by a number of pre- and postsynaptic mechanisms, which have been selected through evolution to keep a tonic and regular activity of 5-HT neurons. As a general rule, antidepressant drugs aim at enhancing serotonergic activity, therefore interfering with the homeostasis of the 5-HT system and activating self-adaptive mechanisms that limit the full antidepressant action [2].

## 2. 5-HT_1A_ Receptor Distribution

While all 5-HT receptor subtypes are localized post-synaptically on 5-HT target cells, the 5-HT_1A_, 5-HT_1B/D_, and 5-HT_2B_ receptors are also localized on 5-HT neurons [6]. 5-HT_1A_ receptors are prFesent in high densities in limbic brain areas (hippocampus, lateral septum), cortical areas (particularly prefronthal and enthorinal cortex), as well as in the raphe nuclei, both dorsal and median [7]. They are being found in the soma, in dendrite, in some cases in the hillock of neurons, and in the cell body and processes of astrocytes [8]. Stimulation of 5-HT_1A_ receptors in prefrontal cortex enhances forebrain catecholamine release, an effect possibly involved in the antidepressant action of the receptor agonists. The physiological release of 5-HT inhibits the activity of PFC pyramidal neurons due to the direct stimulation of 5-HT_1A_ receptors located on the neurons (for a review see [9]).

Presynaptically, the 5-HT_1A_ receptor is the major somatodendritic autoreceptor on the soma and dendrites of serotonergic neurons, where it acts as a “brake” to inhibit the activity of the entire 5-HT system and is thought to delay the antidepressant response [10]. Local release of 5-HT in the raphe nuclei from axonal collaterals or crosstalk between different 5-HT neurons may diminish neuronal firing and produce a negative feedback regulation of transmitter release and may add an extra level of topographical specification. Consistent with their role in regulating serotonergic tone, activation of autoreceptors limits the initial increase of the extracellular 5-HT levels induced by selective serotonin reuptake inhibitors (SSRIs), and delay their therapeutic response [11,12].

**Figure 1:**
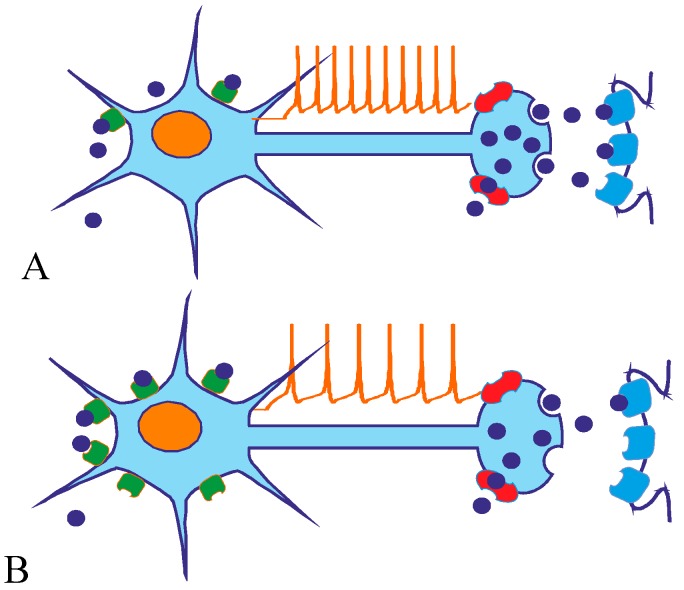
Raphe neurons with low (**A**) and high (**B**) 5-HT_1A_ receptor density. Mice with high somatodendritic 5-HT_1A_ expression have lower basal firing rate (and elevated behavioral despair level). Blue dots—serotonin, red shape—SERT, green shape—5-HT_1A_ receptor (according to [13,14]).

Richardson-Jones *et al.* obtained transgenic mice with normal (referred as 1A-high) and low 5-HT_1A_ autoreceptor levels. It was shown that compared to 1A-high mice, 1A-low mice have indistinguishable levels of 5-HT_1A_ heteroreceptor expression, but displayed about 30% less autoreceptor expression than did the 1A-high mice. In that model, when the serotonergic system was activated, higher intrinsic 5-HT_1A_ autoreceptor levels resulted in lower raphe firing rate (Figure 1). The obtained data also suggested that, at baseline (*i.e.*, non-stressful conditions), levels of serotonin did not differ between the 1A-high and 1A-low mice [13,14].

Postsynaptic 5-HT_1A_ heteroreceptors are expressed in target areas receiving serotonergic innervation. They are located on non-serotonergic neurons, primarily in the limbic areas, such as on the dendrites and soma of glutamatergic pyramidal neurons, and axon terminals of GABAergic and cholinergic neurons. The heteroreceptors are particularly enriched in the hippocampus, where immunohistochemistry and radioligand binding have demonstrated high receptor levels in the stratum radiatum of CA1 and the granule cell layer of the dentate gyrus, and moderate levels in CA3. 5-HT_1A_ heteroreceptors are also highly expressed in the entorhinal cortex, frontal cortex, and lateral septum, and moderately expressed in the amygdala, superior colliculus, piriform cortex, and interpeduncular nucleus, as well as in several hypothalamic and thalamic nuclei. Activation of 5-HT_1A_ heteroreceptors on these distinct neurons mediates hyperpolarizing response to released serotonin and usually reduces neuronal excitability and firing [10,14].

Aside from 5-HT_1A_, the 5-HT_1B_ receptor is also thought to serve as an autoreceptor. 5-HT_1B_ receptors are expressed in the central nervous system (CNS), concentrated in the basal ganglia, striatum, and frontal cortex. In addition, the receptor may also act as a terminal heteroreceptor controlling the release of other neurotransmitters, such as acetylcholine, glutamate, dopamine, noradrenaline, and γ-aminobutyric acid [15,16]. The receptor is also found on cerebral arteries and other vascular tissues. The putative 5HT_1B_ receptor agonist, anpirtoline, has analgesic and antidepressant-like properties in rodents, and 5-HT_1B_ receptor KO mice were reported to be both highly aggressive and have an increased preference for alcohol [16,17].

Studies investigating the relationship between 5-HT_1A_ and 5-HT_1B_ receptors showed that 5-HT_1A/1B_ knockouts had increased extracellular serotonin in the hippocampus, suggesting that the pairing of SSRI with 5-HT_1A/1B_ antagonist might prove to be a potent treatment for anxiety and depression [18]. 5-HT_1B_ receptor agonist (CP 94253) and antagonist (SB 216641) have been shown to be effective in preclinical models of anxiety and CP 94253 was also effective in the model of depression (forced swim test—FST) [19]. It was also shown that the activation of 5-HT_1B_ heteroceptors induces antidepressant-like effect in mice [20].

## 3. 5-HT_1A_ Receptor Regulated Transcription Pathways

### 3.1. Adenylate Cyclase

**Figure 2:**
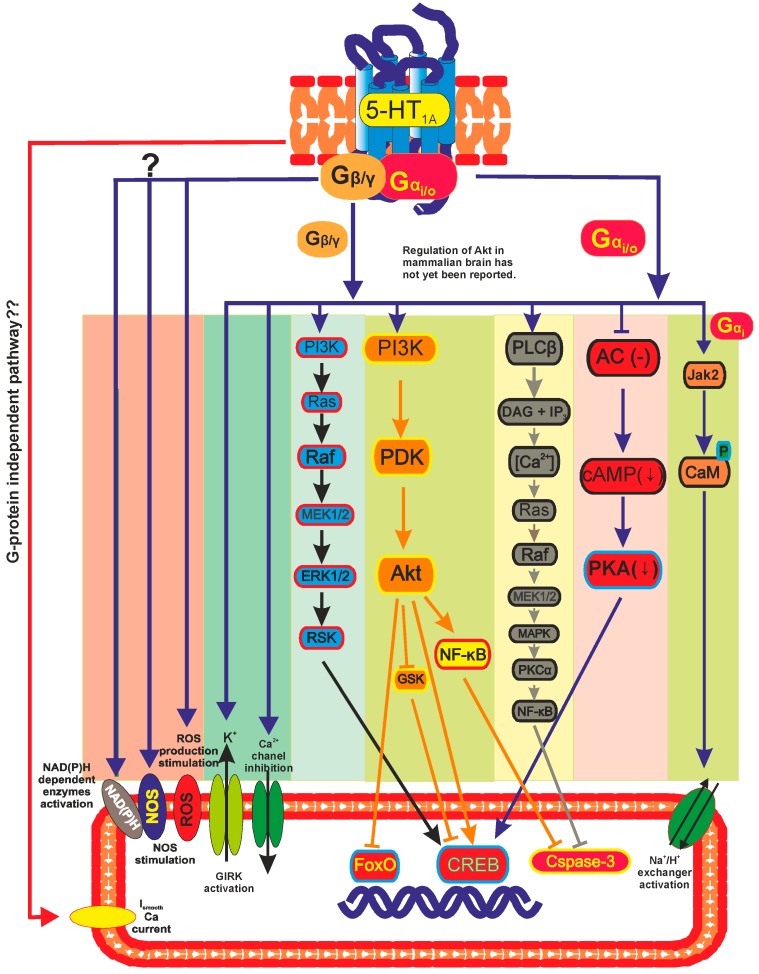
Signal transduction pathways of the 5-HT_1A_ receptor [22,23,24].

The primary coupling linkage of the 5-HT_1A_ receptor (and of all 5-HT_1_ receptors) is to the inhibition of adenylate cyclase (AC) and decrease protein kinase A (PKA) activity. However, the 5-HT_1A_ receptor couples to the broadest panel of second messengers of any of the 5-HT receptors [21]. This receptor has been reported to activate or inhibit various enzymes, channels, and kinases, and to stimulate or inhibit production of diverse soluble second messengers. The receptor has been found to inhibit and activate AC and phospholipase C (PLC), to stimulate nitric oxide synthase (NOS) and a nicotinamide adenine dinucleotide phosphate **(**NADP) oxidase-like enzyme, to activate K^+^ channels and high conductance anion channels, to inhibit Ca^2+^ conductance and inhibit or stimulate Ca^2+^ mobilization, and regulate a number of channels and transporters (Figure 2) [22,23,24]. The 5-HT_1A_ receptor can activate protein kinase C (PKC), Src kinase, and mitogen-activated protein kinases (MAPKs), activate or inhibit phosphatidyl inositol hydrolysis, and stimulate production of reactive oxygen species (both H_2_O_2_ and superoxide) and arachidonic acid [1].

Despite the diversity of second messengers activated, all of the signals are almost completely sensitive to *pertusis* toxin, implicating G_i/o_ proteins in the signals initiated by the 5-HT_1A_ receptor [21]. 5-HT_1A_ heteroreceptors were shown to couple to Gαi-induced inhibition of adenylate cyclase [7,25] while for 5-HT_1A_ autoreceptors the situation is not so clear. Clarke *et al.* [25] found that 8-hydroxy-2-(di-*n*-propylamino)tetralin (8-OH-DPAT) and dipropyl-5-carboxamidotryptamine (5-HT_1A_ receptor agonists) did not inhibit forskolin-stimulated adenylate cyclase activity in raphe region homogenates. On that basis it was accepted that the autoreceptor located in the dorsal raphe nucleus (DRN) does not exhibit coupling to AC-. It was found, however, that also in the raphe nucleus the 5-HT_1A_ autoreceptor may exhibit coupling to Gαi3 and negatively regulate AC. Buspirone, a partial agonist of 5-HT_1A_ receptor, reduced the presynaptic adenylate cyclase activity in raphe nuclei, the effect being reversed by WAY 100,635, the 5-HT_1A_ receptor antagonist. Such behavior was not, however, observed for flibanserin and 8-OH-DPAT (and other 5-HT_1A_ receptor agonists), which did not exhibit coupling to AC activity in raphe nuclei [26,27]. Thus, those ligands exhibited regional ligand-directed receptor trafficking.

Constitutive activity of 5-HT_1A_ receptors has not been extensively examined. Constitutive activity of G-protein (Gαo) has been demonstrated in human 5-HT_1A_ receptors stably expressed in transfected cell lines, as well as in the native rat 5-HT_1A_ receptors in hippocampal membranes, as revealed by the inhibitory effect of inverse agonists, such as spiperone or methiotepin, on basal guanosine-5ʹ-*O*-(3-[^35^S]thio)-triphosphate ([^35^S]GTPγS) binding. In the native rat 5-HT_1A_ receptors, using anti-Gαo-antibody, it was observed that spiperone and methiothepin reduced basal [^35^S] GTPγS binding to Gαo in a concentration-dependent manner to 90% of basal. The inhibition of basal [^35^S] GTPγS binding induced by spiperone and methiothepin was antagonized by WAY 100,635 (5-HT1A selective neutral antagonist) in a concentration-dependent manner, thus indicating that this inverse agonism was mediated by 5-HT_1A_ receptors [28]. In the passive avoidance paradigm, the 8-OH-DPAT-induced decrease in PKA activity in the hippocampus caused increased protein phosphatase-1 activity and a reduction of training-induced phosphorylation of calcium/calmodulin-dependent protein kinase II (CaMKII), and this signaling effect was accompanied by cognitive deficits [29]. Therefore, inhibition of adenylate cyclase and PKA activity may mediate 5-HT_1A_ receptor-regulated behaviors.

### 3.2. GIRK and Calcium Channel

In neurons, activation of the 5-HT_1A_ receptor activates G protein-coupled inwardly-rectifying potassium channels (GIRKs) [30] in the hippocampus [31,32,33] and in the DRN [25,34], an action that profoundly hyperpolarizes neurons and decreases firing [35,36,37,38]. The activation of GIRKs is primarily mediated by G protein βγ subunits upon receptor activation [39,40,41]. The ability of 5-HT_1A_ receptors to activate GIRK-induced hyperpolarizing currents allows them to have a strong effect on neuronal firing and excitability [34], a physiological process that may be linked to 5-HT_1A_ receptor-regulated behaviors [42].

5-HT_1A_ receptor activation also inhibits voltage-gated calcium channel activity to reduce calcium entry [43,44,45]. 5-HT_1A_ receptor-mediated inhibition of Ca^2+^ currents in dorsal raphe was found to be inhibited by a peptide inhibitor of G protein βγ subunit [46].

### 3.3. ERK/MAPK Pathway

**Figure 3:**
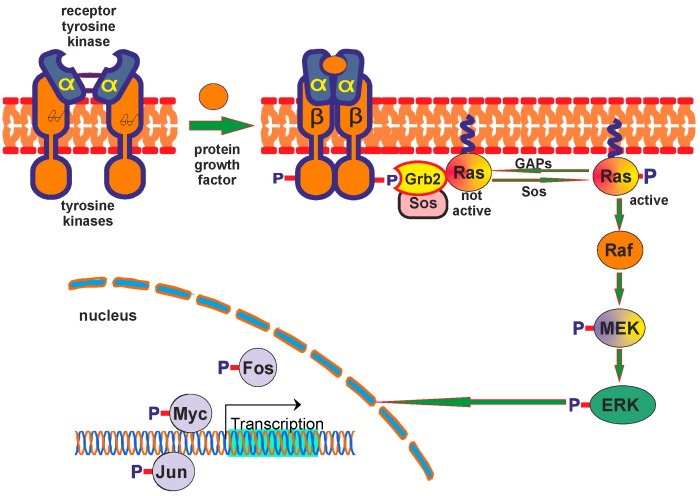
Main elements of the receptor tyrosine kinase pathway.

Another major pathway of the 5-HT_1A_ receptor is by activation of extracellular signal-regulated protein kinase (ERK) (or MAPK), which has been implicated in various aspects of cell proliferation and differentiation [47]. 5-HT_1A_ receptors were first reported to activate ERK by phosphorylation in non-neuronal cells expressing 5-HT_1A_ receptors [48,49]. This effect can be stimulated in transfected human 5-HT_1A_ receptors viaa pathway that shares many of the mediators of growth signals initiated by receptor tyrosine kinases (Figure 3).

The activation pathway involves pertussis toxin-sensitive G protein βγ-subunits, the nonreceptor tyrosine kinase (Src), tyrosine phsphorylated Shc (a molecular docking platform) and a phosphatidylinositol-3 kinase (PI3K) activity [22,48,49,50,51]. Moreover, the pathway appears to involve activation of Sos in a multimolecular complex that likely contains Shc and Grb2, p21*^Ras^,* and p74*^Raf^*^−*1*^ [21,48]. As in growth factor-regulated ERK activation, 5-HT_1A_R-induced ERK activation is mediated by the small GTPases Ras and Raf [22,48,51,52] and active MAPK/ERK kinase (MEK) [22]. Activation of Ras results in sequential activation of Raf1, which in turn phosphorylates and activates MEK 1 and 2. MEK, a direct uspstream protein kinase regulator of ERK, phosphorylates and activates ERK. Additionally, activation of ERK by 5-HT_1A_ receptors in non-neuronal cells can be mediated by the PI3K and phosphatidylcholine-specific PLC in a G protein-dependent manner [22,48,49]. Despite consistent findings in cell systems with heterologous expression of 5-HT_1A_ receptors, effects of 5-HT_1A_ receptors on ERK activity vary in cells of neuronal origin. Consistent findings have shown that 5-HT_1A_ receptor agonists rapidly but transiently increase phosphorylation of ERK in the hypothalamus [53,54,55], and this effect of 5-HT_1A_ receptors is likely an intermediate step for 5-HT_1A_ receptor-induced elevation of oxytocin, adrenocorticotropin (ACTH), and prolactin [53]. In hippocampal-derived differentiated HN2-5 cells, 5-HT_1A_ agonists increase ERK phosphorylation and activity, an effect that is dependent on the small GTPases Ras and Raf, MEK, and calcium mobilization [23,56].

However, this effect of 5-HT_1A_ receptors was not found in the primary culture of hippocampal neurons [57] or fetal rhombencephalic neurons [58]. In differentiated raphe neurons, 5-HT_1A_ receptors are coupled to a Gβγ subunit-dependent decrease in MEK activity and ERK phosphorylation [59]. In the rat hippocampus *in vivo* 5-HT_1A_ receptor activation decreases ERK phosphorylation [54,60,61] and the upstream activator of ERK1/2, phospho-MEK1/2 [61].

The 5-HT_1A_ receptor can regulate a number of ERK-related effectors, including activation of PI3K [48,49], changes in downstream protein kinases, such as the ribosomal S6 kinase (RSK) [62], stimulation of nuclear factor κB (NF-κB) [63], and inhibition of caspase 3 [23,56]. The pathway has been suggested to be involved in neuroprotective mechanisms [23]. ERK may also activate cAMP response element binding (CREB), a widely-studied transcription factor for its gene expression function and the underlying roles in stress, anxiety, and depression, suggesting that ERK signaling pathway may have important impact in mood-related behaviors [64]. The behavioral effects of the MEK/ERK signaling pathway have been reported in several studies, with MEK inhibitors causing diverse behavioral changes in animals, ranging from hyperactivity, reduced or increased anxiety, and depressive-like behavior [65,66,67,68], and MEK inhibitors also block the behavioral effect of antidepressants [69]. It was also demonstrated that the activity of ERK1/2 decreased in the occipital cortex of depressed suicide victims. The MEK/ERK signaling pathways were shown to be involved in transcriptional activation and protein synthesis of neuronal survival and neuroplasticity in depression. Importantly, it was shown that an acute blockade of the MAPK signaling produced a depressive-like phenotype and blocked behavioral actions of antidepressants. Moreover, activation of the MAPK/ERK pathway could inhibit apoptosis by inducing the phosphorylation of Bad (a proapoptotic protein) and increasing the expression of antiapoptotic Bcl-2. Given these observations, MAPK/ERK pathway may be involved in the depression etiopathogenesis and effectiveness of antidepressants [70].

### 3.4. PI3K and Akt Pathway

Another growth factor-regulated signaling pathway, the PI3K and Akt pathway, can also be regulated by 5-HT_1A_ receptors. When tyrosine kinase receptors are activated by growth factors, they recruit PI3K to activate phosphoinositide-dependent kinase (PDK), which phosphorylates and activates Akt [71]. The PI3-K/Akt pathway is classically implicated in the regulation of cell growth, survival, proliferation, and movement [72]. In the mammalian brain, in addition to its functions in neuronal survival and differentiation, several studies have implicated the PI3K/Akt pathway in synaptic plasticity, learning, and memory. It was shown that activation of PI3K/Akt was required for the expression of long-term potentiation (LTP) in the dentate gyrus and CA1 region of the hippocampus. Moreover, pharmacological inhibition of PI3K/Akt significantly impaired the inhibitory avoidance, spatial learning, consolidation, retrieval, and extinction of fear-associated memory [70]. Akt dysregulation has been linked to numerous metabolic diseases including diabetes and obesity, and mental disorders such as schizophrenia and drug abuse. Aberrant Akt function has been progressively linked to a growing number of brain disease states. It has been proposed that Akt may represent a molecular link between diseases driven by insulin resistance (e.g., diabetes) and disorders associated with central monoaminergic disturbances, including depression, schizophrenia and drug abuse [73].

The serotonin receptor class 1 has been reported to activate Erk1/2 and Akt in various cell types [74]. Activation of 5-HT_1A_ receptor in Chinese hamster ovary (CHO) cells resulted in PI3K dependent increased phosphorylation of ERK1/2 and Akt [22]. 5-HT dose-dependently induced activation of Erk1/2 in PC-3, Du145, and LNCaP prostate cancer (PC) cells. Similarly, 5-HT induced phosphorylation of Akt in these cell lines. The action of 5-HT was inhibited to varying degrees by inhibitors of MAPK (U0126) and PI3K (LY294002), as well as by a 5-HT_1A_ receptor antagonist (NAN-190). In addition to proliferation, 5-HT induced migration of PC-3 and Du145 cells, which were alleviated by the aforementioned inhibitors. Regulation of Akt by 5-HT_1A_ receptors in the mammalian brain has not yet been reported; however, some indirect evidence does suggest an effect of 5-HT_1A_ receptors in regulating Akt [24]. It has been shown that 5-HT_1/7_ receptor-selective agonists (5-carboxamidotryptamine and 8-OH-DPAT) can activate Akt in hippocampal primary cultures with similar efficacy and potency as 5-HT, the effects being completely inhibited by the selective 5-HT_1A_ receptor antagonists (p-MPPI and WAY-100635) [57]. It was demonstrated that fluoxetine significantly upregulated expression of the phosphorylated-AKT and ERK1/2 proteins in neural stem cells derived from rats. Besides, expression of phosphorylated-Akt and phosphorylated-ERK1/2 in fluoxetine-treated neural stem cells was effectively blocked by both PI3K inhibitor (LY294002) and MEK inhibitor (PD98059). It was, therefore, suggested that the crosstalk between PI3K/Akt and MAPK/ERK pathways involved Akt and ERK1/2 phosphorylation by fluoxetine treatment [70].

Glycogen synthase kinase 3 (GSK3) is a protein kinase that is primarily phosphorylated and inactivated by Akt [75] and several other protein kinases, such as PKC [76] and PKA [77]. GSK3 is a potential molecular target in several psychiatric disorders, particularly mood disorders, as the mood stabilizer lithium is a selective inhibitor of GSK3 [78,79]. Inhibition of GSK3 by pharmacological or genetic means mimics the effects of antidepressants [80,81] and anti-manic drugs [81,82], whereas impaired regulation of GSK3 results in behavioral abnormalities reminiscent of states of mania and depression [24,83].

Another relevant group of Akt substrates is the Forkhead box O transcription factors (FoxOs). In response to growth factors, active Akt phosphorylates and inactivates FoxOs by exporting them out of the nucleus [84]. In both invertebrate and vertebrate brain, FoxOs can be phosphorylated and inactivated by serotonin via the PI3K/Akt-dependent mechanism [85,86], and the FoxO3a subtype in brain can be inactivated by the antidepressant imipramine [86] and down-regulated by lithium [87]. In addition, mice with FoxO deficiency exhibit antidepressive and anxiolytic behavioral phenotypes [86]. Therefore, regulation of protein substrates by Akt in brain plays a critical role not only in neuronal growth and survival, but also in the maintenance of neuronal activity and behavior.

### 3.5. Na^+^/H^+^ Exchangers

Na^+^/H^+^ exchangers (NHEs) are expressed in the surface of all mammalian cells, serving to regulate cell volume, intracellular pH, and transepithelial transport of Na^+^ and acid-base equivalents. It has been shown that microinjection of activated Ras or transfection of the Ha-Ras oncogene stimulates NHE activity in fibroblasts. The classical effect of GTP-bound Ras is the activation of the ERK1 and ERK2. This is thought to occur primarily through a linear signaling pathway including Ras-GTP → Raf-1 kinase → MEK (MAPK/ERK kinase) → ERK [51]. Activation of 5-HT_1A_ receptor resulted in the formation of a signaling complex that included activation of Janus kinase (JAK2), Ca^2+^/calmodulin (CaM) and NHE-1, and which involves tyrosine phosphorylation of CaM. It was thus proposed that NHE-1 activation proceeded through the pathway involving 5-HT_1A_ receptor → G(i2)α and/or G(i3)α → Jak2 activation → tyrosine phosphorylation of CaM → increased binding of CaM to NHE-1 → induction of conformational change in NHE-1 that unmasks an obscured proton-sensing and/or proton-transporting region of NHE-1 → activation of NHE-1 [88].

### 3.6. Nitric Oxide (NO) Production

The 5-HT_1A_ receptor can also regulate the production of NO in some circumstances. It is supposed that putative 5-HT_1A_ receptor in rat ventral prostate cells can stimulate NOS activity [89]. In contrast, 5-HT_1A_ receptors inhibit *N*-methyl-d-aspartate (NMDA)-induced NO production in the adult rat hippocampus and in human neocortical slices [83,90,91,92] suggesting that the regulation of NO synthesis by 5-HT_1A_ receptor may be complex and cell-specific [21].

Various inhibitors of nitric oxide synthase (NOS) have been shown to exert antidepressant-like behavioral effect in a variety of animal models. Nitric oxide (NO) plays an important role in the brain, and pharmacological manipulations of the NO pathway will constitute a novel approach for therapeutic applications in the future [93,94]. It was recently found that the antidepressant-like effect of TRIM, a nNOS inhibitor, in the rat FST was partially attenuated by the depletion of endogenous serotonin (with the use of *p*-chlorophenylalanine). It was also suggested—on the basis of the experiments with different 5-HT_1_ and 5-HT_2_ receptors antagonists)—that the antidepressant-like effect of TRIM was mediated, at least in part, by an interaction with 5-HT_2_ receptors while non-significant effects were obtained with 5-HT_1_ receptors [95].

### 3.7. Diversity of 5-HT_1A_ Pathways

As already noticed, the 5-HT_1A_ receptor has been found to regulate several channels and transport processes, as well as to activate AD in transfected cells and native tissues (for a review see [21]). In addition, a G protein-independent pathway of 5-HT_1A_ receptor coupling to a smooth inward current (preceding calcium-dependent chloride current) I_smooth_ in *Xenopus laevis* oocytes was also suggested [96].

Raymond *et al.* recently addressed the question about the reasons behind the multiplicity of 5-HT_1A_ receptor signaling. They suggested the ability to activate overlapping pools of G-proteins, activation of distinct signals emanating from G-protein α- and βγ-subunits, different repertoires of G-proteins and signaling enzymes expressed in cells, ratio of G-protein/effector/and receptor, ligand-specific effects, differential rates of desensitization, and cellular compartmentalization, to name just a few. It was suggested that the variables that affect the multiplicity of signaling from single receptor types are not likely to be manifested as “all or none” effects. The specific coupling of single types of 5-HT receptors to signaling pathways is likely to result from the summation of effects specific to the host cell milieu. One other theoretical possibility is that some signals emanating from 5-HT_1A_ (or other 5-HT) receptors may be regulated by effector pathways activated through non-G-protein-activated pathways [1].

## 4. 5-HT1A Receptors and Depression

### 4.1. Serotonin and Depression

Serotonergic system has been implicated in affective illnesses, depression being induced by inhibitors of 5-HT synthesis [97] and tryptophan-depleting diets [98] and being alleviated in the response to SSRIs [99]. Acute tryptophan depletion has been shown to trigger relapse in recovered depressed patients and elicits a depressed mood in normal subjects, while most antidepressant treatments, including SSRIs, increase 5-HT neurotransmission either directly or indirectly. Further, chronic administration of paroxetine in rats with tryptophan depleted levels reduced depression-like behavior [100]. The effects of citalopram have been reported in several studies using rodent in behavioral assays, such as the tail suspension test (TST) and the FST, in which immobility is interpreted as an expression of behavioral despair or entrapment. In the TST, acute administration of citalopram dose-dependently reduced immobility of several mouse strains. In the FST, chronic administration of fluoxetine, another SSRI, reversed the depressive-like behavior by decreasing immobility and increasing escape attempts of rats [101]. Similar effects were observed following fluoxetine directly infused within the raphe nuclei of mice [102]. Increases in the expression of a particular micro-RNA, named miR-16, has been detected following infusion of fluoxetine in the raphe nuclei, mimicking antidepressive-like behavior [102]. Interestingly, deep brain stimulation of the subthalamic nucleus in naive or dopamine-depleted rats decreased the firing rate of serotonergic neurons in DRN and enhanced immobility in the FST, which was counteracted by chronic administration of citalopram [103].

### 4.2. Role of Pre- and Postsynaptic 5-HT_1A_ Receptors

Various 5-HT receptor knockout strains have been developed to study the role of serotonin in modulating the stress response. 5-HT_1A_ knockout mice exhibit anxiety-like behaviors, lower hypothalamic-pituitary-adrenal (HPA) response rates, and have reduced adrenal gland weight [104]. Consistently, a transgenic line overexpressing the murine 5-HT_1A_ receptor in the central nervous system under control of its endogenous promoter had reduced anxiety. The transgenic mice revealed typical phenotypic changes indicative of 5-HT_1A_ receptor overactivity—a reduced molar ratio of 5-hydroxyindoleacetic acid to 5-HT in several brain areas and elevated serotonin levels in the hippocampus and striatum [105]. Additionally, mice with complete 5-HT_1A_ receptor knockout (KO), lacking both auto- and heteroreceptors through life, consistently showed increased anxiety in conflict-based anxiety paradigms tasks, while exhibiting decreased behavioral despair in response to stress (higher mobility time in the FST and TST), suggesting that the absence of 5-HT_1A_ receptors could result in an “antidepressant-like” effect [14,106,107,108,109]. Since behavioral despair in response to stress is decreased by acute treatment with a number of drugs used to treat depression, this phenotype has been referred to as “antidepressed” [13].

Günther *et al.* [110] examined the impact of postsynaptic 5-HT_1A_-receptor overexpression in corticolimbic areas (which are thought to be part of the neuronal circuitry of depression [111]) of male and female mice (with female mice displaying higher receptor binding in the distinct brain areas) on the performance in the FST and suggested the involvement of postsynaptic 5-HT_1A_-receptors in the effects of SSRIs. In the FST untreated male, but not female, overexpressing mice showed an antidepressant-like behavior compared to wild-type mice. Citalopram yielded an antidepressant effect without influencing locomotor activity in overexpressing male but not in wild-type mice. Reboxetine (norepinephrine reuptake inhibitor—NRI) had no antidepressant-like effect in overexpressing mice, but a sex dependent effect in WT mice (antidepressant-like response in female mice). The two partial agonists, buspirone and S 15535, produced no antidepressant-like activity in both genotypes and sexes, but gave aberrant motor effects [110].

It was shown in the transgenic mice model, allowing to independently assess the function of 5-HT_1A_ autoreceptors and heteroreceptors, that suppression of endogenous heteroreceptors was not sufficient to impact anxiety-like behavior [14,112]. Furthermore mice lacking 5-HT_1A_ heteroreceptors throughout the life displayed decreased mobility in the FST, or increased behavioral despair, in adulthood [14]. Suppression of the 5-HT_1A_ heteroreceptors during development leads to an increased behavioral despair in adulthood. In contrast, this phenotype was not observed when heteroreceptor suppression was initiated in adulthood, suggesting that 5-HT_1A_ heteroreceptors act developmentally to establish the circuitry underlying the behavioral response to forced swim stress without affecting conflict-based anxiety paradigms [112]. It was thus concluded that suppression of 5-HT_1A_ heteroreceptors results in behavioral despair, but not anxiety, while ectopic overexpression in the forebrain of this receptor during development rescues the anxious phenotype of whole brain 5-HT_1A_ KO mice [14] (Table 1).

Richardson-Jones *et al.* [13] elaborated a murine model in which it was possible to specifically modulate 5-HT_1A_ autoreceptor levels without affecting heteroreceptor levels. Transgenic strains with high (1A-high) and low (1A-low) 5-HT_1A_ autoreceptor densities were obtained. 1A-high (but not 1A-low) mice displayed the expected dose-dependent hypothermic response to the 5-HT_1A_ agonist, 8-OH-DPAT. Between mice with low and high 5-HT_1A_ autoreceptor levels no differences in the open field paradigm and the light/dark choice test were observed. Additionally, between 1A-low and 1A-high mice no differences in the TST and FST were observed. However, in the FST 1A-high, but not 1A-low, displayed progressively less mobility or more behavioral despair, upon re-exposure on the second day. Following four weeks of repeated stress, 1A-high and 1A-low mice remained indistinguishable in their total exploration in the open field. However, 1A-high, but not 1A-low mice, displayed less mobility in FST and TST. Thus, while decreasing adult levels of 5-HT_1A_ autoreceptors does alter either conflict-based anxiety or behavioral response to an acute stressor, it results in increased physiological reactivity to stress and appears to elicit a more active response to a repeated stress in a depression-related task (reduced depression-like behavior) [13]. The 1A-low, but not 1A-high, responded to 26 days of fluoxetine treatment in the novelty suppressed feeding paradigm (measuring the latency of a mouse to consume food placed in the middle of a brightly lit, aversive arena). On this basis it was suggested that a decrease in 5-HT_1A_ autoreceptor levels in adulthood, prior to antidepressant treatment, is sufficient to confer responsiveness to fluoxetine in an otherwise treatment-resistant population [13]. It was shown that loss of autoreceptors impacts anxiety in the adult, suggesting that the anxious-like phenotype of the 5-HT_1A_ KO mouse likely results from increased serotonergic signaling from a disinhibited raphe [13,14,112]. Donaldson *et al.* found that decreases in 5-HT_1A_ autoreceptors during development (postnatal days P14-P30, with a maximal reduction of 40% at P21 and return to regular levels by P30) leads to a long-term increases in anxiety levels and decreases levels of social engagement, but does not alter depression-like behaviors [113]. Similarly, it was shown that suppression of endogenous 5-HT_1A_ autoreceptors throughout life is sufficient to increase anxiety-like behavior in the adult [112] and does not impact behavior in the FST in adulthood [14]. However, modulating 5-HT_1A_ autoreceptors in adulthood does not impact anxiety-like behavior and results in lower levels of depression-related behaviors [13,114] (Table 1).

On the basis of their results [14] Garcia-Garcia *et al.* suggested the distinct roles of the two endogenous receptor populations mediating anxious or depression-like phenotypes with autoreceptors impacting the establishment of anxiety-like behavior and with heteroreceptors affecting behavior in the FST, a depression-related test [14]. Different roles of pre- and postsynaptic 5-HT_1A_ receptors was suggested by Albert *et al.* who suggested that reduced activity of post-synaptic 5-HT_1A_ receptor is implicated in anxiety, while an increased transcription of 5-HT_1A_ autoreceptor associates with depression and resistance to chronic SSRI treatment [115]. Consistently, reduced expression of presynaptic 5-HT_1A_ receptors - without altering postsynaptic 5-HT_1A_ receptors was sufficient to evoke antidepressant-like effects in mice [13,116,117] (Table 1).

**Table 1:** Relationship between 5-HT_1A_ receptor density and behavioral effects.

Behavioural Effect	Auto- and Heteroreceptors	Autoreceptors	Heteroreceptors
**Whole life knockout** [13,14,104,106,107,108,109,112]			
Anxiety	Elevated	Elevated	No impact
Depression	Lower	No impact	Elevated
**Knock-down between 14 and 30 days (40%)** [113]			
Anxiety	- *	Long-term increase	-
Depression	-	No effect	-
**Adulthood** [9,13,114]			
Anxiety	-	No effect	-
Depression	-	Diminished	-
**Reduced activity**			
Anxiety	-	-	Elevated [115]
Depression	-	Diminished [13,116,117]	-
**Overexpression**			
Anxiety	Diminished [105,118]	-	-
Depression	-	Elevated [110]	-
**Increased transcription**			
Depression	-	Increased [115]	-

* No data.

**Figure 4:**
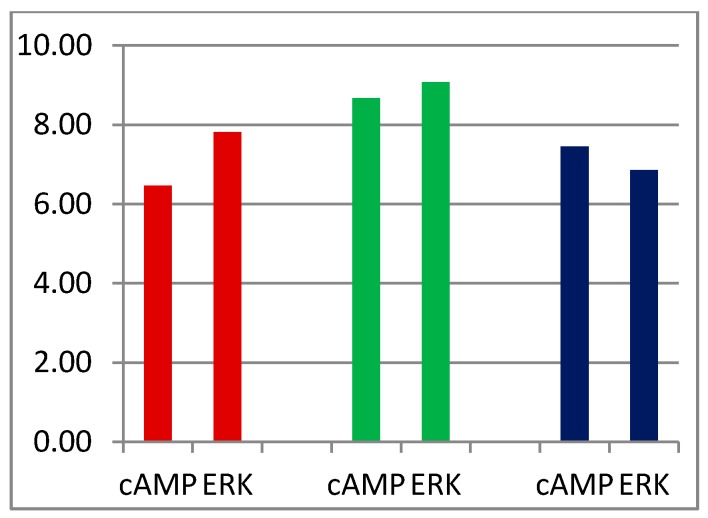
Pharmacological profile (pEC_50_ for cAMP inhibition and ERK1/2 phosphorylation on the *Y* axis) of F15599 (brown), F13714 (green), and 5-HT (dark blue) [55].

An interesting conclusion may be drawn from two novel 5-HT_1A_ receptor agonists F15599 [55] and F13714 [119]. Detailed analysis of their *in vitro*, *in vivo*, electrophysiological, and neurochemical profile indicate that, contrary to F13714, F15599 preferentially activates post- *versus* pre-synaptic 5-HT_1A_ receptors [120] and influences frontal cortex pyramidal neuron electrical activity at doses that are an order of magnitude lower than those that inhibit raphe neuron electrical activity [121]. The compounds exhibited a potent (similar) activity in the FST and in conditioned stress-induced ultrasonic vocalization in rats, despite the fact that F13714 had an *in vitro* potency *ca.* two orders of magnitude greater than F15599 (K_i_ 5-HT_1A_ CHO cells 10.40 and 8.57, respectively) and substantial activity of F13174 at presynaptic 5-HT_1A_ receptors [122]. Additionally, F15599 had a remarkable capacity (as compared to F13714) to reverse phencyclidine (non-competitive antagonist of NMDA-glutamatergic receptors) induced memory/cognition deficits and had a lower propensity than F13714 to induce serotonergic syndrome (lower lip retraction, forepaw treading, flat body posture) [122]. F15599 exhibited lower than F13714 efficacy for ERK phosphorylation (pEC_50_ 7.81 and 9.07, respectively) and cAMP accumulation inhibition (pEC_50_ 6.46 and 8.67, respectively). Both compounds activated G_αi_ and G_αo_ subunits in HeLa-h5-5-HT_1A_ cell membranes, although with different efficacy. It was, thus, suggested that distinct signaling profiles (Figure 4) and functional selectivity for specific receptor activation responses of F15599 underlined its favorable pharmacological profile [55]. It is, however, to be determined which transduction pathway—Activation of pre- or post-synaptic 5-HT1A receptors, ERK phosphorylation, cAMP inhibition, G_αi_ or G_αo_ activation—Is connected to a particular behavioral syndrome. Interestingly, activation of presynaptic 5-HT_1A_ receptors does not influence F13174 antidepressant activity, as compared to F15599.

### 4.3. Antidepressant Treatment and 5-HT_1A_ Receptors

According to pharmacological studies, pre-synaptic and post-synaptic 5-HT_1A_ receptor activation appears to be involved in anxiolytic and antidepressant effects, respectively [9]. Alterations in 5-HT_1A_ receptor levels are commonly observed in depressed individuals. Reduced somatodendritic and postsynaptic 5-HT_1A_ receptor numbers or affinity have been reported in some post-mortem studies of suicide victims, a result consistent with well-replicated positron emission tomography (PET) analyses demonstrating reduced 5-HT_1A_ receptor binding potential in diverse regions such as the dorsal raphe, medial PFC, amygdala, and hippocampus [123].

Post-synaptic 5-HT_1A_ receptors are reduced in several cortical regions in depression and anxiety, while 5-HT_1A_ autoreceptors are increased in depression. Elevated 5-HT_1A_ autoreceptor expression would tend to reduce the activity of 5-HT neurons, while reduced post-synaptic 5-HT_1A_ receptors would result in a blunted behavioral response to 5-HT [10,14]. Hence, post-mortem and genetic studies have shown that individuals with elevated density or activity of 5-HT_1A_-autoreceptors were more susceptible to mood disorders and suicide, and respond poorly to antidepressants [124,125,126]. However, PET studies using [^11^C]-WAY-100635 to label 5-HT_1A_ receptors have shown no case-control differences, increases, nor decreases of the binding potential of 5-HT_1A_ receptors in major depressive disorder (MDD) patients. A lowered 5-HT_1A_ receptor binding potential has also been found in recovered major depressives compared to controls, which has led to the suggestion that a low 5-HT_1A_ receptor density may confer vulnerability to MDD [2]. In this regard, MDD may be associated with a reduced post-synaptic 5-HT_1A_ receptor-mediated function, as suggested by PET scan studies [122,127], although this abnormality persists after clinical remission [128].

Rodent studies have shown that pre-synaptic 5-HT_1A_ (and to a lower extent, 5-HT_1B_) autoreceptors play a major detrimental role in antidepressant treatments, due to the activation of negative feedback mechanisms operating in 5-HT neurons following the increase in extracellular 5-HT evoked by SERT blockade. Hence, excitatory inputs increase 5-HT release in the midbrain raphe. The excess 5-HT activates dendritic 5-HT_1A_ autoreceptors, hyperpolarizes 5-HT neurons and, thus, opposes the incoming excitatory inputs. Antidepressant drugs evoke a pharmacological overactivation of this physiological feedback mechanism by markedly increasing extracellular 5-HT in the raphe nuclei (which contain the largest SERT density in rodent and human brain). Thus, the indirect 5-HT_1A_ receptor activation by SSRI and selective NRI (and by monoaminooxidase inhibitors) reduces serotonergic activity and 5-HT release in forebrain, thereby attenuating the 5-HT elevation produced by SERT blockade in corticolimbic networks thus limiting the activation of postsynaptic 5-HT receptors responsible for clinical antidepressant effects [2,129]. Recent observations with PET suggest the existence of a similar negative feedback mechanism in primate and human brains. Thus, a single, clinically-relevant dose of escitalopram increased 5-HT release in the raphe nuclei and reduced it in the projection areas [130].

This negative feedback mechanism is involved in the delayed action—and possibly, limited efficacy—of antidepressant drugs. The preferential blockade of presynaptic 5-HT_1A_ receptors by pindolol (mixed β-adrenoceptor/5-HT_1A_ receptor antagonist) accelerates the clinical effects of SSRIs [131,132,133]. Pindolol partially blocks the SSRI-induced, 5-HT_1A_ receptor-mediated negative feedback and augments forebrain 5-HT release, mimicking the 5-HT_1A_ autoreceptor desensitization produced by chronic antidepressant treatment [134].

Chronic treatment with antidepressant drugs desensitizes pre-synaptic 5-HT_1A_ autoreceptors, which reduces the efficacy of the autoreceptor-mediated negative feedback and enables a normalization of 5-HT release, thus allowing a greater activation of postsynaptic 5-HT receptors responsible for clinical antidepressant action.

Studies in rats treated chronically with SSRIs have shown an initial decrease of raphe firing at the beginning of treatment, with firing rates recovering to baseline following chronic treatment and 5-HT_1A_ autoreceptor desensitization [135]. The long time required for SSRIs’ clinical efficacy onset (several weeks) may be attributed to the necessity of 5-HT_1A_ autoreceptor desensitization or/and to the time required to evoke plastic changes in certain brain areas involving protein synthesis or trophic actions. Additionally, repeated treatment with 5-HT_1A_ agonists desensitizes pre-synaptic 5-HT_1A_ receptors in the raphe nuclei, which disengages 5-HT neurons from autoreceptor-mediated inhibition. As a consequence, 5-HT neurons are activated by chronic treatment with 5-HT_1A_ agonists, and this counteracts the 5-HT deficit in depression [136,137]. 5-HT_1A_ antagonist can improve the efficacy of SSRIs by either blocking inhibitory 5-HT_1A_ autoreceptors or could have antidepressant-like activity by triggering post-synaptic 5-HT_1A_ receptors and/or producing a faster desensitization of 5-HT_1A_ autoreceptors. In line with this, the selective inactivation of presynaptic 5-HT_1A_ receptors by genetic or molecular means (siRNA) is sufficient to evoke robust antidepressant-like effects in rodents [9].

Several 5-HT_1A_ receptor agonists show antidepressant activity in various animal models, such as TST and FST [135,138]. 5-HT_1A_ receptor KO mice display increased anxiety-related behavior, which, unlike in their wild-type counterparts, cannot be rescued with antidepressant drug treatment [123]. The activation of post-synaptic hippocampal 5-HT_1A_ receptors plays an important role in MDD. Chronic antidepressant treatments tonically activate hippocampal 5-HT_1A_ receptors [123,135]. Furthermore, genetic studies indicate that post-synaptic 5-HT_1A_ receptors are sufficient for antidepressant-like effects in rodents [112]. Antidepressant effects of some 5-HT_1A_ drugs may be attributed to the activation of post-synaptic 5-HT_1A_ receptor sites, an effect likely linked to their therapeutic action [139,140]. Lithium, valproate, SSRIs, tricyclic antidepressants (TCAs), and other treatments, such as electroconvulsive shock therapy, all increase post-synaptic 5-HT_1A_ receptor signaling through either direct or indirect effects [123]. These observations, together with the antidepressant properties of 5-HT_1A_ agonists in preclinical tests, have led to the suggestion of post-synaptic 5-HT_1A_ agonists as a new class of antidepressants [135]. Most 5-HT_1A_ agonists developed so far—and in particular, the azapirones—show full agonist properties at pre-synaptic 5-HT_1A_ autoreceptors and are partial agonists at post-synaptic 5-HT_1A_ receptors, which results in a reduced tone on post-synaptic 5-HT_1A_ receptors after the administration of 5-HT_1A_ agonists [2]. Under conditions of increased serotonin release, the occupancy of post-synaptic 5-HT_1A_ receptors by a partial agonist may actually reduce 5-HT_1A_ receptor transmission by blocking the direct effects of serotonin while inducing only a partial agonist effect instead [122].

Despite the initial hopes placed on the clinical use of 5-HT_1A_ receptor agonists as antidepressant drugs, these agents achieved little clinical success, partly due to their limited clinical efficacy and to the widespread occurrence of gastrointestinal side effects. Several azapirones, such as buspirone, gepirone, ipsapirone or tandospirone, showed antidepressant-like efficacy in preclinical studies, and clinical efficacy in open-label or placebo-controlled trials. The failure of azapirones in MDD may be related to their preferential presynaptic activity [135,141]. Classical 5-HT_1A_ receptor agonists, and particularly azapirones (buspirone, gepirone, *etc.*) are full agonists at raphe 5-HT_1A_ receptors and partial agonists at post-synaptic hippocampal 5-HT_1_A receptors [142]. However, some new agents with chemical structures different from the azapirones (e.g., F15599) display preferential agonist actions at post-synaptic 5-HT_1A_ receptors [55,143].

5-HT_1A_ receptor antagonists may improve the clinical effects of antidepressants by preventing the 5-HT_1A_ autoreceptor-mediated negative feedback [144]. The antagonists may thus mimic the 5-HT_1A_ autoreceptor desensitization effect of chronic SSRIs administration, thus augmenting the effect of SSRIs on forebrain extracellular 5-HT concentration. In this respect, selective antagonists, preferentially blocking pre-synaptic *vs.* post-synaptic 5-HT_1A_ sites, would be required [2,145,146].

It was suggested [9] that 5-HT_1A_ partial agonism, combined with 5-HT reuptake inhibition, can produce antidepressant-like effects. These observations have led to the development of new antidepressant drugs blocking the serotonin transporter (SERT) and having partial agonist activity at 5-HT_1A_ receptors, in order to evoke a higher increase in extracellular 5-HT: Vilazodone, approved in 2011 by the FDA for use in the treatment of MDD, and vortioxetine (LuAA21004, in development) [147,148]. Similarly flibanserin, DU-125530, and OPC-14523 have reached late stage clinical development as antidepressant drugs [9].

Mayorga *et al.* showed that fluoxetine and paroxetine failed to ameliorate immobility behavior in 5-HT_1A_ KO mice exposed to a stressor, suggesting that 5-HT_1A_ receptor activation is a necessary feature of the antidepressive response [149]. It was, however, shown that 5-HT_1A_ KO mice respond to TCAs, but not to the SSRI fluoxetine, in the TST and the novelty-suppressed feeding test, suggesting that the 5-HT_1A_ receptors are a critical component in the mechanism of action of SSRIs, but not TCAs [148,150]. It should also be noted that according to clinical data, full 5-HT_1A_ blockade neither enhances nor cancels the antidepressant effect of fluoxetine in MDD patients [139], suggesting the involvement of other 5-HT receptors (e.g., 5-HT_4_ receptor) [151].

Recent studies suggested that neurogenesis is involved in the action of antidepressants [152]. Neurogenesis, the process of neuronal stem cell proliferation, differentiation and survival, has also been thought to occur in humans, at a slow, but detectable rate [150]. Santarelli *et al.* [153] demonstrated the involvement of antidepressants in adult hippocampal neurogenesis. In addition, this study showed that 5-HT_1A_ knockout mice did not exhibit neurogenesis or respond behaviorally to SSRI treatment, implicating these receptors in antidepressant-mediated neurogenesis. The antidepressant effects of 5-HT_1A_ receptor stimulation may be related to the induction of hippocampal neurogenesis, a common feature of antidepressant treatments [154], which depends on 5-HT_1A_ receptor activation [150], but also the functional remodeling of corticolimbic circuits involved in MDD [122]. It was thus suggested that the treatment with antidepressants could lead to adaptive changes such as neurogenesis over a period of weeks, which may account for the delayed therapeutic effect of these drugs [155].

As with the 5-HT_1A_ receptors, acute SSRI administration activates terminal 5-HT_1B_ receptors, thus reducing 5-HT synthesis and release. Likewise, chronic administration of SSRI also results in desensitization of terminal 5-HT_1B_ autoreceptors, suggesting that plasticity in both the 5-HT_1A_- and 5-HT_1B_-mediated autoregulatory function may be important in the therapeutic profile of SSRI [156,157]. Moreover, as observed with 5-HT_1A_ receptors, the administration of 5-HT_1B_ receptor antagonists augments the neurochemical and behavioral effects of SSRI [158,159]. The effects of SSRIs have also been analyzed in genetic rodent models of depressive-like behaviors, such as in mice lacking the 5-HT_1B_ receptor. The acute administration of citalopram or paroxetine failed to decrease the immobility in the FST of the 5-HT_1B_ (KO) compared to WT mice, suggesting that antidepressant effects of SSRI depend on activation of the 5-HT_1B_ receptor [6]. Interestingly, the effects of 5-HT_1B_ antagonists on pre-synaptic 5-HT function are additive to those of 5-HT_1A_ antagonists, indicating a diversity of mechanisms to augment the effects of SSRI [160]. Mice lacking both 5-HT_1A/1B_ autoreceptors exhibit an increased 5-HT transmission also associated to anxiety-like behavior. While acute paroxetine failed to reverse anxiety-like behavior, its chronic administration still led to anxiolytic effects in the elevated plus maze [6].

## 5. Models of Depression Involved Circuits

The current limitation of SSRI/SNRI treatments most likely derives from the poor knowledge of the pathophysiology of major depression, in common with other psychiatric disorders. The lack of a unifying theory on the cause of depression, together with the partial success of antidepressant drugs enhancing serotonergic (and to a lesser success, noradrenergic) function, has dominated the antidepressant drug development so far. Numerous clinical and preclinical studies indicate that disturbances in serotonergic activity may be associated with major depression. Noradrenergic and dopaminergic neurotransmission has also been implicated, although the exact changes in these monoamine systems are unknown. Historically, a functional hypoactivity of these systems has been assumed in depression. This view was mainly based on the observation that antidepressant drugs increase monoaminergic function (particularly serotonin and norepinephrine). One commonality of these ascending monoaminergic systems is that their cell bodies are located in the brain stem and that their activity is tightly controlled by the PFC, a cortical area where metabolic abnormalities have been reported in depressive patients. Thus, the raphe nuclei, the locus coeruleus, and the ventral tegmental area, where the cell bodies of ascending serotonergic, noradrenergic, and dopaminergic neurons are located, respectively, receive dense afferents from dorsal and ventral parts of the medial PFC in rodents, which are equivalent to dorsal and ventral cingulate are as in primate and human brain. The treatment with antidepressant drugs would restore monoamine function in cortical and limbic areas, thus improving depressive symptoms. Based on these, Artigas has shown a schematic representation (not included) of the functional connectivity between the medial prefrontal cortex and the dorsal and median raphe nuclei of the midbrain [2].

**Figure 5:**
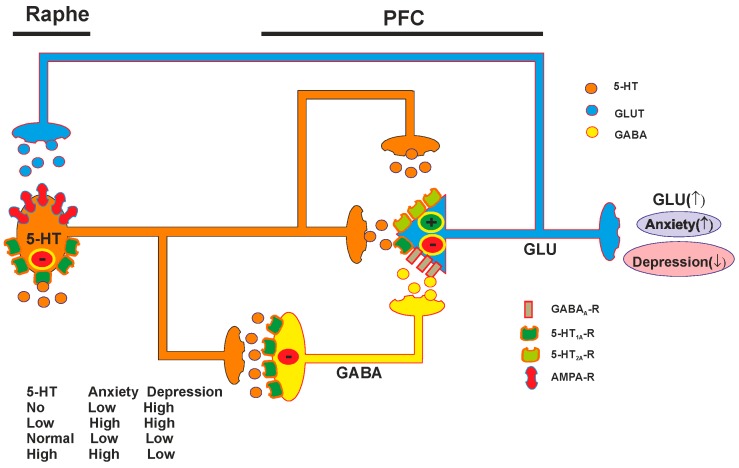
Adult 5-HT raphe-PFC circuitry in anxiety and depression model (according to Albert *et al.*) [161]. The model shows 5-HT neurons (brown) projecting to prefrontal cortex GABAergic interneurons (yellow) and glutamatergic pyramidal neurons (blue) with transmitter release illustrated as small circles of the same colors. Receptors: 5-HT_1A_ (green), 5-HT_2A_ (light green), α-amino-3-hydroxy-5-methyl-4-isoxazolepropionic acid (AMPA)-glutamate (purple) and GABA_A_ receptors (blue). The response in the target neurons (red ovals): stimulatory (+), inhibitory (−). (↑)—elevation, (↓)—decrease. 5-HT_1A_ heteroreceptors and GABA_A_ receptors reduce the activity of the pyramidal neurons while 5-HT_2A_ receptors activate the neurons.

Albert *et al.* proposed a simplified model of raphe and PFC circuitry involvement in anxiety and depression (Figure 5) [161]. It was suggested that there are two major sub-populations of heteroreceptors in PFC: 5-HT_1A_ receptors on pyramidal glutamatergic neurons and on GABA interneurons that inhibit the activity of both neuron types [162]. In rodent PFC, ~50% of pyramidal neurons and ~25% of GABAergic interneurons express 5-HT_1A_ receptors [163], and this proportion is even higher (up to 80%) in upper cortical layers of primate and human PFC [162]. It should also be noted that 5-HT_1A_ receptors are abundantly coexpressed (in *ca.* 80%) with excitatory 5-HT_2A_ receptors in rodent PFC [161]. 5-HT_2A_ receptors have 10-fold lower affinity for 5-HT and with maturation 5-HT_1A_ receptor inhibition predominates over 5-HT_2A_ excitatory function. In this model the activity of the GABA interneurons exhibits inhibitory activity on glutamatergic pyramidal neurons while 5-HT_1A_ heteroreceptors diminish the GABAergic activity. In the anxiety model, under normal conditions both 5-HT_1A_ heteroreceptors on pyramidal and interneurons are engaged, resulting in a balance between 5-HT_1A_-mediated inhibition and dys-inhibition on pyramidal neurons. On the basis of evidence from human imaging studies, it was suggested that different sub-populations of PFC neurons with different targets mediate anxiety *vs.* depression behavior. Due to these differences, the serotonin circuitry model for depression involves similar components but is slightly different from the anxiety model. First, opposite to anxiety, activation of the pyramidal neuron is associated with reduced depression and increased resilience. Second, it was proposed that the 5-HT_2A_ receptor is weakly active in the mature pyramidal neurons controlling depression. The model predicts elevated anxiety at the low and high serotonin levels and elevated depression at the lack of serotonin and its low levels [162].

The model was shown to fit with the genetic model where mutants with reduced or eliminated 5-HT show depression phenotypes, while suppression of 5-HT_1A_ autoreceptors in adults to increase the 5-HT activity reduces depressive behavior. Reduced interneuron 5-HT_1A_ receptors would enhance interneuron firing and inhibit pyramidal firing, leading to a depression phenotype similar to the low or no 5-HT condition. Suppression of pyramidal 5-HT_1A_ heteroreceptors (using the CaMKIIα promoter to target pyramidal neurons) would be predicted to increase pyramidal neuron firing and display an anxiety phenotype rather than depression. Over-expression of 5-HT_1A_ autoreceptors would reduce 5-HT activity leading to a hyperactive anxiety circuit by preferentially relieving inhibition of pyramidal neurons. On the other hand, global knockout of all 5-HT_1A_ receptors would allow for hyper-activation of raphe neurons (due to absence of 5-HT_1A_ autoreceptor) and activation of stimulatory pyramidal 5-HT_2A_ receptors that is not antagonized by pyramidal 5-HT_1A_ receptors, leading to increased anxiety. Pyramidal 5-HT_2A_ receptor activation may also actively suppress interneuronal GABA-mediated inhibition via activation of protein kinase C. Consistent with a pro-anxiety role, mice lacking 5-HT_2A_ receptors display reduced anxiety that is reversed by cortical re-expression of 5-HT_2A_ receptors [162].

**Figure 6:**
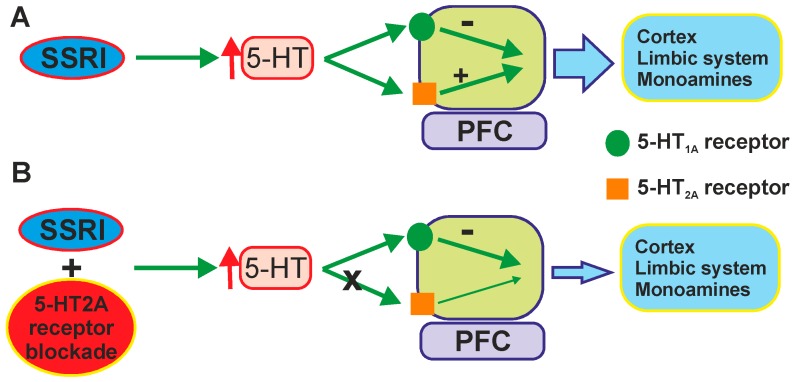
The potential mechanism underlying the augmenting effects of 5-HT_2A_ receptor blockade. (**A**) Increased extracellular 5-HT concentration may activate limbic monoamine system; (**B**) Blockade of activating 5-HT_2A_ receptors may reduce a potential hyperactivity of the monoamine system (according to [2]).

It was shown that numerous antidepressants and antipsychotic drugs bind with relatively high affinity at 5-HT_2A_ receptors and several clinical studies have shown that atypical antipsychotics augment the clinical response in treatment-resistant patients. The effect was suggested to be due to their ability to occupy 5-HT_2_ receptors, and more specifically to block 5-HT_2A_-mediated response. The increased extracellular 5-HT concentration produced by SERT blockade activates excitatory 5-HT_2A_ receptors and inhibitory 5-HT_1A_ receptors (Figure 6A). Given the large co-expression of 5-HT_1A_ and 5-HT_2A_ receptors in the neocortex [164] blockade of 5-HT_2A_ receptors (or atypical antipsychotic drugs) blocks excitatory effects of 5-HT on pyramidal neurons. That may enhance inhibitory 5-HT_1A_ receptor-mediated neurotransmission in cortical and limbic areas, an effect likely linked to the antidepressant activity (Figure 6B). This effect may reduce a potential hyperactivity in ventral cingulate areas and normalize the functional connectivity between ventral and dorsal PFC areas and with limbic areas and monoamine systems [2].

Bevilacqua *et al.* have suggested a functional interaction between SERT and the 5-HT_2B_ receptor—a receptor implicated in 5-HT-dependent phenotypes, including impulsivity, aggressivity and suicidality [165]. *Ex vivo* studies have indicated that 5-HT_2B_ receptors might participate in the control of SERT in raphe neurons [166], while *in vivo* studies further confirmed that 5-HT_2B_ receptors contribute to the behavioral and physiological effects of the SERT-targeting 5-HT releasers, 3,4-methylenedioxymethamphetamine (MDMA, the club-drug ecstasy) and dexfenfluramine [167,168,169]. Diaz *et al.* [170] reported that the acute response to SSRIs in the FST, a classical test for antidepressant activity, is absent in mice lacking 5-HT_2B_ receptors. Conversely, a 5-HT_2B_ receptor agonist induced an antidepressant-like action in the FST, suggesting that this receptor is required for acute SSRI effects and might modulate serotonergic tone. It was also shown that long-term behavioral and neurogenic SSRI effects were abolished after either genetic or pharmacological inactivation of 5-HT_2B_ receptors. Conversely, direct agonist stimulation of 5-HT_2B_ receptors induced an SSRI-like response in behavioral and neurogenic assays suggesting that antidepressant effects of SSRI depend on activation of the 5-HT_2B_ receptor [169,171].

Another candidate for mediating the antidepressant-like effects of SSRIs is the 5-HT_2C_ receptor. 5-HT_2_C receptor agonists show antidepressant-like effects in multiple animal models (both acute and chronic) of depression [172]. For example, 5-HT_2C_ receptor agonists decrease immobility time and increase swimming time in the FST in rats in a manner comparable to SSRIs. The effects of the 5-HT_2C_ receptor agonists and SSRIs in the rat forced swim test are antagonized by the 5-HT_2C_ antagonists [173] consistent with the role for 5-HT_2C_ receptors in mediating antidepressant-like effects of 5-HT_2C_ receptor agonists and SSRIs (recently Rosenzweig-Lipson *et al.* have shown that novel 5-HT_2C_ receptor agonist WAY-163909 exhibited antidepressant-like effects in rodents).

## 6. Conclusions

5-HT_1A_ receptors have, for long time, been considered as interesting targets for antidepressant drugs. It was thus postulated that postsynaptic 5-HT_1A_ agonists could form a new class of antidepressant drugs. It was also stated that most 5-HT_1A_ agonists developed so far—and in particular, the azapirones—show full agonist properties at pre-synaptic 5-HT_1A_ autoreceptors and are partial agonists at post-synaptic 5-HT_1A_ receptors, which results in a reduced tone on post-synaptic 5-HT_1A_ receptors after the administration of 5-HT_1A_ agonists.

There is a, more or less, consistent view concerning the role of 5-HT_1A_ receptors in depression and anxiety, with autoreceptors impacting the establishment of anxiety and heteroreceptors affecting depression-like tests. Results with 5-HT_1A_ receptor KO animals have shown that 5-HT_1A_ receptor KO mice exhibit increased anxiety-like behavior and decreased levels of depression. In line with that 5-HT_1A_ receptor overexpressing mice exhibit reduced anxiety. It was shown that 5-HT_1A_ autoreceptor down-regulation resulted in antidepressant-like effects, and increased transcription was associated with depression, resistance to chronic SSRIs treatment, and mice lacking heteroreceptors throughout life exhibited increased levels of depression.

It was suggested that the anxious-like phenotype of the 5-HT_1A_ KO mouse likely results from increased serotonergic signaling from a disinhibited raphe. While decreasing adult levels of 5-HT_1A_ autoreceptors does alter either conflict-based anxiety or behavioral response to an acute stressor, it may result in increased physiological reactivity to stress and appears to elicit more active responses to a repeated stress in a depression-related task (reduced depression-like behavior).

It was shown, that although 5-HT_1A_ KO mice do not respond to the SSRI fluoxetine, they respond to TCAs (TST and the novelty-suppressed feeding test), suggesting that the 5-HT_1A_ receptors are a critical component in the mechanism of action of SSRIs, but not TCAs. According to clinical data, full 5-HT_1A_ blockade neither enhances nor cancels the antidepressant effect of fluoxetine in MDD patients suggesting the involvement of other 5-HT receptors (e.g., 5-HT_4_ receptor).

Agonist-directed trafficking for 5-HT_1A_ receptor signaling exists in rat native brain tissue. Thus, the receptors have the ability to activate overlapping pools of G-proteins, activate distinct signals emanating from G-protein α- and βγ-subunits and differing repertoires of G-proteins and signaling enzymes expressed in cells. It may express different ratios of receptor/G-protein/effector, ligand-specific effects, differential rates of desensitization, and cellular compartmentalization, as well as activate effector pathways through non-G-protein-activated pathways. The human 5-HT_1A_ receptors, located in different brain regions despite similar [3H]-8-OH-DPAT binding profiles, present a different functional pharmacology. Moreover, receptor trafficking appears different at pre- and post-synaptic sites, with pre-synaptic 5-HT_1A_ receptors showing more marked adaptive processes, including desensitization and down-regulation [156,174,175].

The 5-HT_1A_ receptor may still be considered as valuable target for antidepressant drugs. New mixed 5-HT_1A_ receptor ligands/SERT inhibitors seem to possess interesting pharmacological profiles. Although identifying the real molecular and brain-specific target for 5-HT_1A_ receptor ligands may be a very complicated task due to the receptor signal transduction pathway complexity, it may result in better targeting, raising hope for more effective medicines for various pathologies.

It should also be noted that 5-HT_1A_ receptor cooperates with other transduction systems (like 5-HT_1B_ and 5-HT_2A/2B/2C_ receptors, GABA, glutamine) with relying its antidepressant and/or anxiolytic activity. Most probably additional serotonergic targets like 5-HT_1B_, 5-HT_2B_ or 5-HT_4-7_ receptors should also be considered. 5-HT_1B_ receptor antagonists administered alone or with antidepressants have been shown to be effective in preclinical models of depression; the activation of 5-HT_1B_ heteroceptors induces antidepressant-like behavior. As with the 5-HT_1A_ receptors, acute SSRI administration activates terminal 5-HT_1B_ receptors, thus reducing 5-HT synthesis and release. Likewise, chronic administration of SSRI also results in desensitization of terminal 5-HT_1B_ autoreceptors, suggesting that plasticity in both the 5-HT_1A_- and 5-HT_1B_-mediated autoregulatory function may be important in the therapeutic profile of SSRI. Mice lacking both 5-HT_1A/1B_ autoreceptors exhibit an increased 5-HT transmission also associated to anxiety-like behavior. 5-HT_2B_ receptors are also expressed by raphe serotonergic neurons. SSRI-induced increase in hippocampal extracellular 5-HT concentration is strongly reduced in the absence of functional 5-HT_2B_ receptors and selective 5-HT_2B_ agonists may mimic SSRI responses. 5-HT_2B_ receptors were shown to be required for the therapeutic actions of SSRIs. 5-HT_4-7_ receptors are also considered as new molecular targets for antidepressant drugs.
